# The Effect of Cannabis Use Disorder on Mortality and Other Outcomes in Asthma: A Nationwide Analysis (2016-2021)

**DOI:** 10.7759/cureus.94969

**Published:** 2025-10-20

**Authors:** Samuel Sule-Saa, Pyae Phyo Hein, Jeffrey A Sackey, Daniel Pinkrah, Muhanned Towfig, Anusha Akella, Rebecca Kotei, Richard DiCasoli, Andleeb Sherazi, Kalpana Panigrahi

**Affiliations:** 1 Internal Medicine, Interfaith Medical Center, Brooklyn, USA; 2 Medicine, One Brooklyn Health, Brooklyn, USA; 3 Medicine, Interfaith Medical Center, Brooklyn, USA; 4 Internal Medicine, Ridge Hospital, Accra, GHA; 5 Internal Medicine, Brookdale University Hospital Medical Center, Brooklyn, USA; 6 Critical Care, One Brooklyn Health, Brooklyn, USA; 7 Internal Medicine, One Brooklyn Health, Brooklyn, USA

**Keywords:** acute asthma, cannabis use disorder (cud), mortality, severe asthma exacerbation, status asthmaticus

## Abstract

Background

Asthma, a chronic respiratory disease affecting over 300 million individuals globally, significantly impacts the quality of life and healthcare systems. In recent years, cannabis use disorder (CUD) has emerged as a potential complicating factor. Cannabis, though increasingly legalized and perceived as benign, poses risks to respiratory health. This study explores the relationship between CUD and asthma outcomes, focusing on mortality and morbidity trends from 2016 to 2021 in a nationwide context.

Objective

The objective of this study is to evaluate the impact of CUD on in-hospital mortality, severe asthma exacerbations, and healthcare resource utilization among patients hospitalized for asthma.

Methods

A retrospective cohort analysis of nationwide asthma admissions between 2016 and 2021 was conducted using the Nationwide Inpatient Sample database. A total of 552,160 cases were stratified into CUD and non-CUD cases. A logistic regression analysis was used to examine the association between CUD and in-hospital mortality as well as the severity of exacerbations among asthmatic patients. We utilized linear regression models to assess the impact of CUD on total charges and length of stay for asthma admissions, adjusting for demographic and hospital-level confounders.

Results

Of the 552,160 asthma hospitalizations, 4.2% (N = 23,300) of patients had comorbid CUD. CUD patients were younger (a mean age of 35.3 years compared to 51.4 years in non-CUD patients) and predominantly male. Our study showed the odds of in-hospital mortality were significantly greater in patients with CUD (adjusted OR 2.40, 95% CI 1.62-3.55, p < 0.01). CUD was associated with increased odds of severe asthma exacerbations/status asthmaticus (OR 2.39, 95% CI 1.92-2.99, p <.01). However, the adjusted odds ratio (OR) was attenuated (adjusted OR 1.35, 95% CI 1.07-1.71, p = 0.012). Total hospital charges were significantly higher in the CUD group after adjustment (coefficient 2091.31, 95% CI 660.71-3521.9, p = 0.004). There was no significant difference in length of stay between groups (adjusted coefficient -0.06, 95% CI -0.1-0.06, p = 0.7).

Conclusion

Patients hospitalized for asthma with CUD are associated with higher mortality, increased risk of severe asthma exacerbations, and higher hospital charges. These findings underline the need for targeted interventions in asthmatic patients with CUD to improve clinical outcomes and reduce healthcare burden.

## Introduction

Asthma, a common chronic respiratory disease, affects over 260 million individuals worldwide as of 2021 and 8.7% of the population in the United States [1,2]. It remains a serious global health problem, contributing to substantial morbidity and mortality. According to the Centers for Disease Control and Prevention (CDC), about 3517 people died from asthma in 2021 in the United States [3]. Asthma significantly impacts healthcare system expenditures, accounting for $81.9 billion in total economic costs from 2008 to 2013 in the United States [4]. While advancements in asthma management have reduced morbidity and mortality from asthma, emerging lifestyle factors and comorbidities continue to impact optimal outcomes. Exposure to allergens and irritants is a well-known trigger for asthma exacerbation. Identifying potential triggers has been a cornerstone of asthma management, enabling adequate disease control and preventing exacerbations. A recent systematic review and meta-analysis suggests a significant association between cannabis use and greater odds of having asthma [5]. In that regard, cannabis use emerges as a potential risk factor for asthma.

Cannabis was first legalized for medical use in the state of California in 1996 through the Compassionate Use Act of 1996 [6]. In 2012, Colorado and Washington became the first states to legalize recreational use. With cannabis legalization expanding across jurisdictions and public perception shifting toward its recreational and medicinal benefits, concerns regarding its effects on the respiratory system have become important topics of discussion. The prevalence of cannabis use has been on an increasing trend, with reported cannabis use in those aged 12 or older jumping from 11% in 2002 to 21.9% in 2022 [7].

The link between asthma and cannabis use disorder (CUD) raises significant concerns regarding the potential adverse effects of cannabis on respiratory health. While some individuals may use cannabis to alleviate asthma symptoms [8], consumption of cannabis by smoking produces many of the same harmful constituents as tobacco smoking, with some in greater quantities [9]. This inhalation can lead to increased airway inflammation, bronchial hyperreactivity, and heightened risk of asthma exacerbations. Furthermore, the psychoactive properties of cannabis may contribute to altered perceptions of symptom severity, potentially leading individuals to underestimate the seriousness of their condition or delay necessary medical intervention. Moreover, cannabis use is associated with endothelial dysfunction, as with tobacco smoking [10,11]. As smoking remains the primary means of consuming cannabis, its effects on health are quite concerning, and understanding the impact of CUD on asthma outcomes is crucial, as it may influence treatment strategies and patient education.

Despite increasing clinical awareness, there is still limited data on the nationwide impact of CUD on asthma-related outcomes. As the prevalence of CUD grows alongside the increased acceptance of cannabis use, it is essential to examine these relationships to ensure that healthcare providers can offer informed guidance tailored to the unique needs of asthmatic patients. This study examines the relationship between CUD and key clinical results for patients hospitalized with asthma in the United States. We analyze data from the Nationwide Inpatient Sample between 2016 and 2021 to determine whether CUD affects in-hospital mortality rates, the frequency of severe asthma exacerbations, and healthcare resource utilization. By clarifying these connections, our findings aim to improve clinical care and inform public health strategies for managing asthma in an era of rising cannabis use.

## Materials and methods

Study design and data source

This retrospective cohort study analyzed data from the National Inpatient Sample (NIS), part of the Healthcare Cost and Utilization Project (HCUP), from 2016 to 2021. The NIS represents the largest publicly available all-payer inpatient healthcare database in the United States, containing discharge data from approximately 20% of all U.S. hospital admissions [12].

Study population

The study cohort included all patients aged 18 years and older with a primary diagnosis of asthma. These patients were then stratified into two groups based on the presence or absence of concomitant CUD. The International Classification of Diseases, Tenth Revision, Clinical Modification (ICD-10-CM) codes were used to identify asthma and CUD diagnoses. Exclusions included pediatric patients and incomplete demographic data.

Variables and outcomes

Patient demographics included age at admission, gender, race/ethnicity, and geographic location, based on the patient's ZIP code residence. Socioeconomic status was assessed using median household income quartiles for the patient's ZIP code. Hospital characteristics included geographic region, bed size, and teaching status, while comorbidity burden was quantified using the Charlson Comorbidity Index.

The primary outcomes included in-hospital mortality and severe asthma exacerbation/status asthmaticus; secondary outcomes comprised total hospital charges and length of stay (LOS). Severe asthma exacerbation was defined using specific ICD-10-CM codes for status asthmaticus and acute severe asthma requiring intensive management.

Statistical analysis

Descriptive statistics were calculated for all variables, with continuous variables reported as means with standard deviations and categorical variables as frequencies and percentages. Chi-square tests were used for categorical variables, and t-tests were used for continuous variables to compare groups.

Multivariable logistic regression models were constructed to assess the association between CUD and binary outcomes (mortality and severe exacerbation/status asthmaticus), with results reported as odds ratios (ORs) with 95% confidence intervals (CI). Linear regression models were used for continuous outcomes (total charges and LOS), with results reported as coefficients with 95% CI.

All multivariable models were adjusted for potential confounders, including age at admission, gender, race, median household income quartile, patient residence, Charlson Comorbidity Index score, hospital location/teaching status, hospital bed size, and hospital region. Both unadjusted and adjusted analyses were performed. Statistical significance was set at p < 0.05. All analyses were conducted using appropriate survey weights to account for the complex sampling design of the NIS.

## Results

In our nationwide analysis of asthma admissions from 2016 to 2021, we observed a total of 552,160 cases. Of these, 530,795 were classified as severe asthma exacerbation or status asthmaticus. Notably, 4.3% (N = 22,905) of the patients had a co-diagnosis of CUD.

Patient and hospital characteristics

According to Table 1, CUD patients were younger, with a mean age of 35.3 years, compared to 51.4 years in non-CUD patients. A higher proportion of males were observed in the CUD group (50.4% vs. 26.6%), with the majority of patients in the CUD group being black (46.7%).

A greater percentage of CUD patients resided in central metropolitan counties with populations of ≥1 million (47.7% vs. 38.9%), and the median annual income in patients' ZIP codes was lower in the CUD group, with 45.1% residing in areas with incomes of <$38,999.

The majority of CUD patients were admitted to large hospitals (44.9%) and urban teaching facilities (78.3%, p < 0.01). The regional distribution of hospital admissions was relatively evenly spread across the Northeast, Midwest, South, and West. CUD patients had higher Charlson Comorbidity Index scores (82.6% vs. 59.5%), with the majority of CUD patients also being on Medicare insurance (53.1% vs. 29.2%).

**Table 1:** Patient sociodemographic and hospital characteristics CUD: cannabis use disorder

Patient characteristics	Non-CUD	CUD	p-value
No. (%) of asthma admissions	528,860 (95.8)	23,300(4.2)	<0.01
No. (%) of severe asthma/status asthmaticus	507,971 (95.7)	22,905(4.3)	<0.01
Mean age at admission, in years	51.4	35.3	-
Gender no. (%)			
Male	140,677 (26.6)	11,743 (50.4)	<0.01
Female	388,183 (73.4)	11,557 (49.6)	<0.01
Race/ethnicity, no. (%)			
White	235,343 (44.5)	7,433 (31.9)	<0.01
Black	169,235 (32.0)	10,881(46.7)	<0.01
Hispanic	87,262 (16.5)	3,658 (15.7)	<0.01
Asian or Pacific Islander	14,808 (2.8)	256 (1.1)	<0.01
Native American	3,702 (0.7)	163 (0.7)	<0.01
Other	17,717 (3.35)	909 (3.9)	<0.01
Location of patient’s residence, no. (%)			
Central counties of metro areas ≥ 1 million population	205,727(38.9)	11,114 (47.7)	<0.01
Fringe counties of metro areas ≥ 1 million population	134,330 (25.4)	4,730 (20.3)	<0.01
Counties in metro areas 250,000-999,999 population	96,780 (18.3)	4,264 (18.3)	<0.01
Counties in metro areas 50,000-249,999 population	38,078 (7.2)	1,724 (7.4)	<0.01
Micropolitan counties	32,260 (6.1)	979 (4.2)	<0.01
Not metropolitan or micropolitan counties	21,682 (4.1)	489 (2.1)	<0.01
Charlson Comorbidity Index score, no. (%)			
1	314,672 (59.5)	19,246 (82.6)	<0.01
2	116,349 (22.0)	2,493 (10.7)	<0.01
3	97,839 (18.5)	1,561(6.7)	<0.01
Median annual income in patient’s ZIP code, US$, no. (%)			
1-38,999	196,207 (37.1)	10,508 (45.1)	<0.01
39,000-47,999	131,157 (24.8)	5,452 (23.4)	<0.01
48,000-62,900	114,234 (21.6)	4,287 (18.4)	<0.01
>63,000	87,262 (16.5)	3,052 (13.1)	<0.01
Insurance type, no. (%)			
Medicaid	185,101 (35.0)	2,470 (10.6)	<0.01
Medicare	154,427 (29.2)	12,372 (53.1)	<0.01
Private	142,792 (27.0)	4,753 (20.4)	<0.01
Uninsured	46,540 (8.8)	3,705 (15.9)	<0.01
Hospital characteristics			
Hospital region, no. (%)			
Northeast	139,090 (26.3)	6,291 (27.0)	<0.01
Midwest	103,128 (19.5)	4,893 (21.0)	<0.01
South	199,380 (37.7)	6,897 (29.6)	<0.01
West	87,262 (16.5)	5,219 (22.4)	<0.01
Hospital bed size, no. (%)			
Small	132,215 (25.0)	5,545 (23.8)	<0.01
Medium	167,120 (31.6)	7,293 (31.3)	<0.01
Large	229,525 (43.4)	10,462 (44.9)	<0.01
Teaching status of hospital, no. (%)			
Rural	42,309 (8.0)	1,095 (4.7)	<0.01
Urban non-teaching	116,878 (22.1)	3,961 (17.0)	<0.01
Urban teaching	369,673 (69.9)	18,244 (78.3)	<0.01

Outcomes

The in-hospital mortality rate for patients admitted for asthma was 0.35%. We conducted logistic regression analyses to examine the association between CUD and mortality in asthma admissions. Both unadjusted and adjusted models were employed, with the latter accounting for various demographic and hospital-level confounders. The adjusted logistic regression analysis revealed a higher odds of mortality in asthmatic patients with CUD (adjusted OR 2.40, 95% CI 1.62-3.55, p < 0.01), as shown in Table 2.

Also, a similar logistic regression models were applied to investigate the relationship between CUD and the occurrence of severe exacerbation or status asthmaticus in asthma patients. CUD was associated with an increased odds of severe asthma exacerbations/status asthmaticus (OR 2.39, 95% CI 1.92-2.99, p < 0.01); however, the adjusted OR was attenuated but still statistically significant (aOR 1.35, 95% CI 1.07-1.71, p = 0.012).

We utilized linear regression models to assess the impact of CUD on total charges and length of stay for asthma admissions. Both unadjusted and adjusted analyses were performed, and Table 2 shows that the total hospital charges were significantly higher in the CUD group after adjustment (coefficient 2,091.31, 95% CI 660.71-3,521.9, p = 0.004). There was, however, no significant difference in length of stay between groups (adjusted coefficient -0.06, 95% CI -0.1-0.06, p = 0.7).

**Table 2:** Adjusted and unadjusted regression models of mortality, severe exacerbation, total hospital charges, and length of stay in asthma admissions in patients with comorbid cannabis use disorder vs. without cannabis use disorder p < 0.05 indicates statistical significance. Multivariable regression models were adjusted for age at admission, gender, race, median household income, national quartile for patient ZIP code, Charlson Comorbidity Index, location/teaching status of the hospital, region of the hospital, and patient’s residence.

Outcome	Model	Effect measure	Estimate	95% CI	p-value
In-hospital mortality	Unadjusted	Odds ratio	2.33	1.64 - 3.31	<0.01
	Adjusted¹	Odds ratio	2.4	1.62 - 3.55	<0.01
Severe exacerbation/status asthmaticus	Unadjusted	Odds ratio	2.39	1.92 - 2.99	<0.01
	Adjusted1	Odds ratio	1.35	1.07 - 1.71	0.012
Total hospital charges	Unadjusted	Coefficient ($)	28.22	-1,368.72 - 1,425.15	0.97
	Adjusted¹	Coefficient ($)	2,091.31	660.71 - 3,521.9	0.004
Length of stay (days)	Unadjusted	Coefficient	-0.52	-0.60 - -0.45	<0.01
	Adjusted¹	Coefficient	-0.06	-0.1 - 0.06	0.7

**Figure 1:**
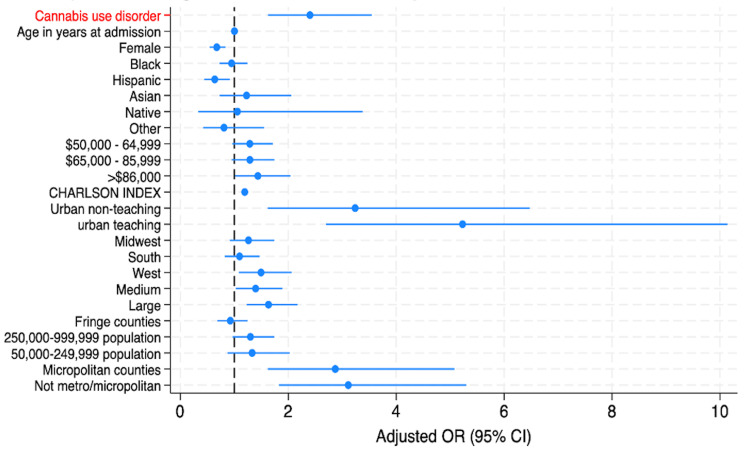
Forest plot of adjusted odds ratios for in-hospital mortality among patients with asthma admissions from 2016 to 2021 The vertical dashed line at an odds ratio of 1.0 represents the line of no effect. Confidence intervals that cross this line are not statistically significant.

The forest plot in Figure 1 indicates that CUD was a significant predictor of increased inpatient mortality in asthma admissions. Other predictors of in-hospital mortality were comorbid disease burden, hospital-related factors, such as large, urban hospitals and population size. Conversely, factors associated with a statistically significant reduction in mortality risk included female sex and Hispanic ethnicity.

## Discussion

This nationwide study evaluated the impact of CUD on asthma hospitalizations from 2016 to 2021. Among over 552,000 cases, 4.2% had CUD. CUD patients were notably younger, were more often male and Black, and more likely to reside in low-income urban areas and be treated at large, urban teaching hospitals. These demographic findings are consistent with national epidemiologic data, which show that CUD is more prevalent among younger adults, males, and Black individuals, and is associated with lower socioeconomic status and urban residence [13-16]. Racial and social class discrimination have been identified as important contributors to cannabis initiation and progression to CUD among Black youth and young adults, further compounding disparities in asthma outcomes [17].

CUD was independently associated with adverse outcomes. Adjusted logistic regression showed significantly higher odds of in-hospital mortality in patients with CUD (aOR 2.40, 95% CI 1.62-3.55). Additionally, CUD was associated with significantly higher hospital charges (adjusted coefficient $2,091.31), though no difference in length of stay was observed. These findings are in line with prior studies demonstrating that CUD is associated with increased asthma morbidity, higher resource utilization, and greater risk of severe exacerbations, including status asthmaticus [18-21]. For example, a recent meta-analysis found a pooled OR of 1.31 for the association between cannabis use and asthma diagnosis, and large-scale electronic health record analyses confirm that regular cannabis use increases the risk of asthma and other respiratory diseases, independent of tobacco use [18,19]. States with legalized recreational or medical cannabis have experienced 14-20% higher asthma admission rates, with increased costs primarily affecting Medicare and privately insured populations [18].

The association between CUD and status asthmaticus is supported by mechanistic and clinical evidence. Inhaled cannabis can precipitate acute asthma attacks and status asthmaticus, particularly in susceptible individuals [21]. The American Academy of Pain Medicine, in its consensus guidelines, notes that cannabis use is associated with increased cough, sputum production, and wheezing, and that these symptoms often resolve with cessation [20]. A dose-response relationship has also been observed, with a higher frequency of cannabis use linked to greater asthma prevalence and morbidity [22]. However, some survey data suggest that not all cannabis users with asthma experience worse control or more frequent exacerbations, indicating heterogeneity in individual risk [23].

Regarding costs and length of stay, the present study’s findings of increased hospital charges but no significant difference in LOS among CUD patients are consistent with national trends. Comorbid substance use and mental illness are associated with higher costs and, in some studies, modestly prolonged LOS [19,20]. The incremental cost burden of CUD is likely multifactorial, reflecting increased severity of illness, higher rates of status asthmaticus, and greater comorbidity burden. Public health implications are substantial.

The rising prevalence of CUD, particularly among socioeconomically disadvantaged and minority populations, may exacerbate existing disparities in asthma outcomes [15-17]. These findings underscore the need for targeted prevention and intervention strategies, including culturally tailored approaches to reduce CUD and its respiratory complications.

In summary, the findings of this study demonstrated that CUD is associated with increased risk of severe asthma exacerbations, including status asthmaticus and inpatient mortality, higher hospital costs, and persistent sociodemographic disparities. The results highlight the importance of screening for CUD in asthma patients, addressing social determinants of health, and implementing public health policies to mitigate the adverse impact of cannabis use on respiratory health.

Several factors limited our study. Firstly, because our study employed a retrospective design, we can only establish an association between CUD and Asthma severity, not causation. Secondly, since our data was taken from the Nationwide Inpatient Sample, which relies mainly on ICD coding, there may be misclassification or underreporting of diagnoses such as CUD or asthma severity, which can affect the reliability of our findings. Additionally, because CUD was treated as a binary variable, with no data on duration, frequency, or method of use, it limits the ability to establish an association between dose and potential outcomes. Lastly, there could be several unmeasured confounding variables that can lead to bias.

## Conclusions

This nationwide analysis provides compelling evidence of the association of CUD with severe asthma outcomes, including status asthmaticus, increased inpatient mortality, higher hospital costs, and persistent sociodemographic disparities. These associations persist even after adjusting for sociodemographic and clinical confounders and are consistently supported by other studies. The results highlight the importance of screening for CUD in asthma patients, addressing social determinants of health, and implementing public health policies to mitigate the adverse impact of cannabis use on respiratory health.

Future research should focus on prospective studies to establish a causal pathway and evaluate the impact of cannabis cessation on asthma outcomes. By integrating clinical vigilance with public health action, we can better mitigate the growing burden of CUD on respiratory health and health equity.
